# Comparative value of novel inflammatory indices in predicting incident carotid atherosclerosis: SIRI outperforms other markers in a general population

**DOI:** 10.3389/fcvm.2026.1760659

**Published:** 2026-02-18

**Authors:** Jun-Long Tao, Yu-Ting Wei, Hai-Feng Zhang, Chang-Chen Liang, Jia-Min Chen, Xiao-Na Wang, Li Cao, Yi Ning, Bo Bi

**Affiliations:** 1 School of Public Health, Hainan Medical University, Hainan Academy of Medical Sciences, Haikou, China; 2 Key Laboratory of Tropical Translational Medicine of Ministry of Education, Hainan Medical University, Hainan Academy of Medical Sciences, Haikou, China; 3 Beijing MJ Healthcare Center, Beijing, China

**Keywords:** carotid atherosclerosis, cohort study, inflammatory indices, systemic inflammation, systemic inflammation response index

## Abstract

**Background:**

Systemic inflammation is a core driver of carotid atherosclerosis (CAS). Although various inflammatory indices derived from routine blood tests have been associated with prevalent CAS, most existing evidence is limited to cross-sectional designs, and a systematic head-to-head comparison of their predictive value is lacking. This study aimed to bridge this gap by evaluating the associations of six composite inflammatory indices with the risk of incident CAS to identify the optimal predictive marker.

**Methods:**

We conducted a prospective cohort study comprising 12,289 participants free of CAS at baseline from a health screening center in Beijing. Six inflammatory indices—Neutrophil-to-Lymphocyte Ratio (NLR), Platelet-to-Lymphocyte Ratio (PLR), Systemic Immune-Inflammation Index (SII), Systemic Inflammation Response Index (SIRI), Aggregate Index of Systemic Inflammation (AISI), and Neutrophil Percentage-to-Albumin Ratio (NPAR)—were calculated based on baseline data. Cox proportional hazards models, restricted cubic splines, and Random Survival Forest (RSF) models were utilized to assess associations and predictive performance. Mediation analysis was performed to investigate the role of Body Mass Index (BMI).

**Results:**

During follow-up, 2,432 (19.8%) participants developed CAS. After multivariable adjustment, all six inflammatory indices were independently associated with an increased risk of CAS. Among them, SIRI demonstrated the most robust predictive value; participants in the highest quartile of SIRI had a 46% higher risk compared to those in the lowest quartile (Hazard Ratio [HR] = 1.46, 95% Confidence Interval [CI]: 1.30–1.64, *P* < 0.001). RSF analysis confirmed the superior predictive importance of SIRI. Notably, a significant sex interaction was observed (*P* for interaction = 0.031), with a more pronounced association found in men. Furthermore, mediation analysis revealed that BMI partially mediated this association.

**Conclusion:**

Our findings identify SIRI as a robust and independent predictor of CAS development, outperforming other common inflammatory indices. As a readily available and cost-effective biomarker, SIRI offers significant potential for enhancing early risk stratification for subclinical atherosclerosis in clinical practice.

## 1 Introduction

Cardiovascular diseases (CVDs) represent the leading cause of morbidity and mortality worldwide, imposing a substantial burden on public health systems (1, 2). Atherosclerosis, the principal underlying pathophysiological process of CVDs, is characterized as a chronic, low-grade inflammatory disease of the arterial wall (3, 4). Carotid atherosclerosis (CAS), serving as a crucial “window” to systemic atherosclerosis, is an independent predictor of future cardiovascular events (5).

Inflammation plays a pivotal role throughout the entire course of atherosclerosis, from initiation and progression to plaque destabilization (6, 7). In recent years, systemic inflammatory indices derived from routine laboratory tests have garnered considerable attention due to their cost-effectiveness, convenience, and wide availability. These indices, such as the Neutrophil-to-Lymphocyte Ratio (NLR), Platelet-to-Lymphocyte Ratio (PLR), Systemic Inflammation Response Index (SIRI), Systemic Immune-Inflammation Index (SII), Aggregate Index of Systemic Inflammation (AISI), and the Neutrophil Percentage-to-Albumin Ratio (NPAR), are designed to capture the complex immuno-inflammatory network. They achieve this by integrating various hematological parameters (e.g., neutrophils, lymphocytes) and, in some cases, biochemical markers like albumin, which reflects both nutritional and anti-inflammatory status (8–10). Although the clinical utility of these indices has been extensively explored beyond oncology—demonstrating robust prognostic value in cerebrovascular and critical care settings (11, 12)—it remains unclear which specific marker offers the superior predictive performance for early-stage atherosclerosis.

Existing evidence, primarily from cross-sectional and case-control studies, has preliminarily established an association between these inflammatory indices and the presence of prevalent CAS (13–15). However, the inherent limitations of these study designs preclude the determination of the temporal sequence between the inflammatory state and the onset of atherosclerosis, thereby providing insufficient evidence for their predictive utility regarding future risk. Although a few cohort studies have begun to explore the association of specific inflammatory markers with incident carotid plaque, there remains a profound lack of large-scale, prospective cohort studies that systematically investigate and compare the associations of multiple novel and conventional inflammatory indices with the future risk of developing CAS (16, 17). Furthermore, given the close interplay between inflammation and metabolic disorders such as obesity, the potential mediating role of Body Mass Index (BMI) in the causal pathway linking these inflammatory indices to CAS risk remains to be elucidated. Therefore, utilizing a large-scale prospective cohort, this study aimed to systematically evaluate and compare the associations of multiple blood cell-derived inflammatory indices with incident carotid atherosclerosis to identify the optimal predictive marker. Additionally, we further explored the dose-response patterns of these associations, their consistency across various subgroups, and preliminarily investigated the potential mediating role of Body Mass Index (BMI).

## 2 Methods

### 2.1 Study population

**Figure 1:**
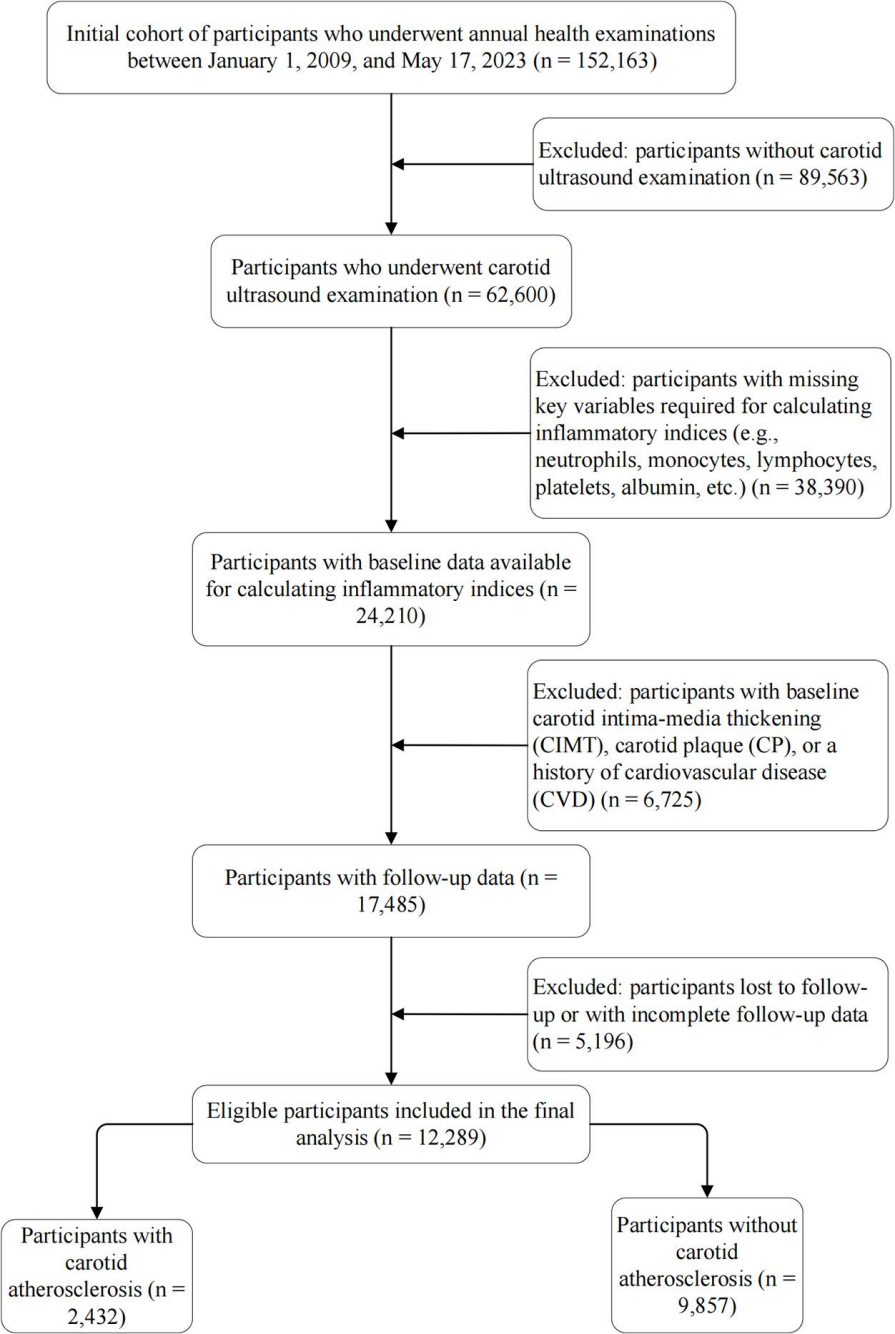
Flowchart of the study population. The diagram illustrates the selection process of eligible participants from the initial cohort. A total of 12,289 participants were included in the final analysis. CIMT, carotid intima-media thickness; CP, carotid plaque; CVD, cardiovascular disease.

This study was based on a prospective open cohort study conducted at the MJ Health Check Center in Beijing using data from January 1, 2009, to May 17, 2023. The initial cohort comprised 152,163 adults. We first excluded 89,563 individuals who did not undergo carotid ultrasound. To address potential selection bias, we compared the baseline characteristics of those with and without ultrasound examinations (Supplementary Table S1). The results showed that the excluded group was comparable to the included population in terms of age (median: 41 vs. 42 years) and the prevalence of hypertension (13.6% vs. 15.2%) and diabetes (5.5% vs. 5.2%), indicating that the lack of ultrasound examination was likely not driven by younger age or lower cardiovascular risk. Subsequently, 38,390 participants with missing key inflammatory biomarkers were excluded. Comparison between the included and excluded groups revealed that while statistical differences existed due to the large sample size, the clinical characteristics were largely similar (Supplementary Table S2). We further excluded individuals with prevalent CIMT thickening, carotid plaque, or CVD (*n* = 6,725), as well as those lost to follow-up (*n* = 5,196). Finally, 12,289 eligible participants were included (Figure 1).

### 2.2 Definition of inflammatory markers

Based on baseline complete blood count (CBC) and serum albumin results, we calculated six systemic inflammatory biomarkers. First, we calculated three dual-component ratios: the neutrophil-to-lymphocyte ratio (NLR), calculated as the neutrophil count divided by the lymphocyte count; the platelet-to-lymphocyte ratio (PLR), calculated as the platelet count divided by the lymphocyte count; and the neutrophil percentage-to-albumin ratio (NPAR), defined as the neutrophil percentage (%) divided by the serum albumin concentration (g/L).

Second, we calculated three more complex composite inflammatory indices: the Systemic Inflammation Response Index (SIRI), calculated as (neutrophil count×monocyte count)/lymphocyte count; the Systemic Immune-Inflammation Index (SII), calculated as (platelet count×neutrophil count)/lymphocyte count; and the Aggregate Index of Systemic Inflammation (AISI), calculated as (platelet count×neutrophil count×monocyte count)/lymphocyte count.

In all the above formulas, the counts of neutrophils, lymphocytes, monocytes, and platelets were expressed in units of ×10^9^/L.

### 2.3 Definition of CAS

The outcome of interest was incident carotid atherosclerosis (CAS), assessed using a high-resolution color Doppler ultrasound system (Sonoscape S50) equipped with a 7.5 MHz linear array transducer. All examinations were performed by experienced sonographers following a standardized scanning protocol to ensure consistent image acquisition. Key vascular segments, including the common carotid artery, carotid bifurcation, and internal carotid artery, were systematically evaluated in both transverse and longitudinal planes.

CAS was defined according to widely accepted criteria as either: (1) the new appearance of at least one carotid plaque, characterized by focal intimal thickening ≥ 1.5 mm, or (2) new carotid intima-media thickening (CIMT) ≥ 1.0 mm during the follow-up period. Participants meeting either of the above criteria were considered to have developed CAS.

To ensure the reliability of the assessment, a random sample of 10% of the participants was selected for quality control. For inter-observer agreement, a senior sonographer independently reviewed the images blinded to the initial results, yielding a Cohen's Kappa of 0.90 for the diagnosis. For intra-observer agreement, to minimize recall bias, the primary sonographer re-assessed the same stored images after a two-week interval, resulting in a Cohen's Kappa of 0.92.

### 2.4 Covariates

Demographic information (age and sex), socioeconomic status (educational level and annual income), and lifestyle factors were collected through a standardized, structured questionnaire. Smoking and alcohol consumption statuses were dichotomized into “current smoker/non-current smoker” and “current drinker/non-current drinker,” respectively.

Anthropometric measurements, including height and weight, were performed using standard protocols, and Body Mass Index (BMI) was calculated as weight in kilograms divided by the square of height in meters (kg/m^2^). Hypertension was defined as a systolic blood pressure ≥140 mmHg, a diastolic blood pressure ≥90 mmHg, or a self-reported history of hypertension. Diabetes mellitus was defined as a fasting plasma glucose level ≥7.0 mmol/L or a self-reported history of diabetes.

Furthermore, venous blood samples were collected from all participants after an overnight fast. Serum levels of total cholesterol (TC), triglycerides (TG), high-density lipoprotein cholesterol (HDL-C), and creatinine, as well as whole blood red blood cell (RBC) count, were measured using an automated analyzer.

### 2.5 Statistical analysis

All statistical analyses were conducted using R software (version 4.5.1). A two-sided *P*-value of less than 0.05 was considered statistically significant for all tests. Baseline characteristics of the study participants were summarized according to the occurrence of CAS. Continuous variables not conforming to a normal distribution were presented as medians with interquartile ranges (IQR) and compared between groups using the Wilcoxon rank-sum test. Categorical variables were described as frequencies and percentages [*n* (%)] and assessed using the Pearson's Chi-squared test.

To evaluate the association between various inflammatory indices and the risk of CAS, we constructed Cox proportional hazards regression models to calculate Hazard Ratios (HRs) and their 95% confidence intervals (CIs). Given the high correlation among the inflammatory indices (e.g., SIRI and AISI), they were included in the Cox models individually rather than simultaneously to avoid multicollinearity. We developed a series of models: Model 1 was an unadjusted model; Model 2 was adjusted for age, sex, and BMI; and Model 3 was further adjusted for the variables in Model 2 plus educational level, hypertension, diabetes mellitus, annual income, smoking status, alcohol consumption status, HDL-C, TG, RBC, creatinine, and TC. Multicollinearity among the covariates in the fully adjusted models was assessed using the Variance Inflation Factor (VIF). The proportional hazards assumption was verified using scaled Schoenfeld residuals. The association strength was also assessed per 1-standard deviation (1-SD) increase in each inflammatory biomarker.

Kaplan–Meier survival curves were generated to visualize the cumulative incidence of CAS across quartiles of the inflammatory indices, with differences between curves evaluated by the log-rank test. To explore potential non-linear relationships, we fitted restricted cubic splines (RCS) with four knots placed at the 5th, 35th, 65th, and 95th percentiles. Spearman correlation analysis was used to assess correlations among the inflammatory indices.

To comprehensively compare the predictive performance of the multiple inflammatory indices, Random Survival Forest (RSF) models were constructed. The variable importance (VIMP) method was employed to rank the inflammatory indices based on their predictive capacity.

To ensure the robustness of our findings, we conducted several additional analyses. Pre-specified subgroup analyses were performed by stratifying the population based on baseline age (<45 vs. ≥45 years), sex, BMI (<24 vs. ≥24 kg/m^2^), hypertension, diabetes mellitus, educational level, annual income, smoking status, and alcohol consumption status. Potential effect modification was tested by introducing a multiplicative interaction term between the inflammatory index and the stratification variable into the fully adjusted Cox model. Furthermore, a sensitivity analysis was performed by excluding participants diagnosed with CAS within the first year of follow-up to mitigate potential reverse causality bias. Finally, to assess the potential impact of unmeasured confounding, E-values were calculated specifically for the optimal inflammatory index identified by the RSF analysis. The E-value quantifies the minimum strength of association that an unmeasured confounder would need to have with both the index and CAS to fully explain away the observed associations, conditional on the measured covariates.

## 3 Results

### 3.1 Baseline characteristics

A total of 12,289 eligible participants were ultimately included in this study. During a median follow-up of 2.15 years [interquartile range (IQR), 1.04–4.18 years], 2,432 (19.8%) participants developed incident carotid atherosclerosis (CAS). Supplementary Table S3 presents the baseline characteristics of the participants, stratified by the occurrence of CAS. Compared to participants without CAS, individuals who developed incident CAS exhibited significantly different characteristics at baseline: they were older (median 48 vs. 41 years), had a higher proportion of males (70.8% vs. 58.4%), a higher BMI (median 24.8 vs. 24.0 kg/m^2^), and a higher prevalence of hypertension (24.8% vs. 12.9%) and diabetes (10.3% vs. 4.0%). Furthermore, the CAS group had higher proportions of current smokers and drinkers, but lower levels of education. All the above differences were statistically significant (*P* < 0.05). Regarding baseline inflammatory biomarkers, the CAS group had significantly higher levels of SIRI (median 0.67 vs. 0.62) and AISI (median 146 vs. 139) than the non-CAS group (*P* < 0.001). Conversely, the levels of SII (median 389 vs. 397) and PLR (median 115 vs. 122) were significantly lower in the CAS group than in the non-CAS group (*P* < 0.001). This seemingly paradoxical observation suggests that the unadjusted associations of SII and PLR with CAS might be masked by confounding factors.

### 3.2 Association of inflammatory indices with incident CAS risk

Before evaluating the associations, we verified the assumptions of the Cox proportional hazards models. The test based on scaled Schoenfeld residuals indicated that the proportional hazards assumption was not violated for any of the inflammatory indices (all *P* > 0.05; Supplementary Table S4). Additionally, collinearity diagnostics for the fully adjusted models showed that all Variance Inflation Factors (VIFs) were less than 5, indicating no severe multicollinearity among the covariates (Supplementary Table S5).

In the multivariable Cox proportional hazards regression models, all evaluated inflammatory indices showed independent associations with the risk of incident CAS after full adjustment (Table 1, Model 3). Among them, the associations for AISI and SIRI were particularly prominent. Compared to the lowest quartile (Q1), participants in the highest quartile (Q4) of AISI had a 63% increased risk of CAS (HR = 1.63, 95% CI: 1.44–1.84), while those in the Q4 of SIRI had a 46% increased risk (HR = 1.46, 95% CI: 1.30–1.64). Both exhibited a significant dose-response trend (all *P* for trend < 0.001). The highest quartiles of NLR, SII, PLR, and NPAR also showed a significant increase in risk, with HRs ranging from 1.21 to 1.28. This multivariable analysis clarified the paradoxical findings from the baseline characteristics.

**Table 1:** Hazard ratios (95% CIs) for the associations between systemic inflammatory indices and incident carotid atherosclerosis.

Variables	Model 1		Model 2		Model 3	
	HR (95% CI)	*P* value	HR (95% CI)	*P* value	HR (95% CI)	*P* value
NLR						
Q1	—		—		—	
Q2	1.19 (1.06, 1.33)	0.002	1.18 (1.06, 1.32)	0.003	1.17 (1.05, 1.31)	0.008
Q3	1.10 (0.98, 1.23)	0.106	1.14 (1.02, 1.28)	0.026	1.12 (1.00, 1.26)	0.056
Q4	1.24 (1.11, 1.39)	<0.001	1.28 (1.14, 1.43)	<0.001	1.28 (1.14, 1.44)	<0.001
Per 1-SD increase	1.06 (1.02, 1.10)	0.002	1.07 (1.03, 1.11)	<0.001	1.08 (1.04, 1.12)	<0.001
SIRI						
Q1	—		—		—	
Q2	1.15 (1.02, 1.30)	0.019	1.14 (1.01, 1.28)	0.036	1.09 (0.96, 1.23)	0.168
Q3	1.35 (1.20, 1.52)	<0.001	1.34 (1.19, 1.50)	<0.001	1.28 (1.14, 1.44)	<0.001
Q4	1.59 (1.42, 1.78)	<0.001	1.55 (1.39, 1.74)	<0.001	1.46 (1.30, 1.64)	<0.001
Per 1-SD increase	1.13 (1.09, 1.16)	<0.001	1.14 (1.10, 1.17)	<0.001	1.12 (1.09, 1.16)	<0.001
SII						
Q1	—		—		—	
Q2	1.07 (0.95, 1.19)	0.259	1.17 (1.05, 1.31)	0.006	1.14 (1.02, 1.27)	0.024
Q3	1.05 (0.94, 1.17)	0.399	1.16 (1.04, 1.30)	0.008	1.13 (1.01, 1.26)	0.037
Q4	1.08 (0.96, 1.20)	0.212	1.28 (1.14, 1.44)	<0.001	1.22 (1.09, 1.37)	<0.001
Per 1-SD increase	1.03 (0.99, 1.07)	0.147	1.09 (1.05, 1.13)	<0.001	1.08 (1.04, 1.13)	<0.001
AISI						
Q1	—		—		—	
Q2	1.16 (1.02, 1.31)	0.025	1.19 (1.05, 1.35)	0.008	1.17 (1.03, 1.33)	0.013
Q3	1.30 (1.14, 1.47)	<0.001	1.44 (1.27, 1.64)	<0.001	1.41 (1.24, 1.60)	<0.001
Q4	1.56 (1.38, 1.76)	<0.001	1.70 (1.50, 1.92)	<0.001	1.63 (1.44, 1.84)	<0.001
Per 1-SD increase	1.10 (1.06, 1.13)	<0.001	1.13 (1.09, 1.17)	<0.001	1.11 (1.07, 1.15)	<0.001
PLR						
Q1	—		—		—	
Q2	0.95 (0.85, 1.05)	0.309	1.06 (0.95, 1.18)	0.285	1.10 (0.98, 1.22)	0.093
Q3	0.94 (0.84, 1.05)	0.272	1.12 (1.00, 1.25)	0.055	1.16 (1.04, 1.30)	0.009
Q4	0.94 (0.84, 1.06)	0.316	1.18 (1.05, 1.32)	0.007	1.25 (1.11, 1.41)	<0.001
Per 1-SD increase	0.99 (0.95, 1.03)	0.623	1.08 (1.03, 1.12)	<0.001	1.11 (1.06, 1.16)	<0.001
NPAR						
Q1	—		—		—	
Q2	1.15 (1.02, 1.30)	0.023	1.08 (0.96, 1.20)	0.214	1.10 (0.97, 1.24)	0.141
Q3	1.16 (1.02, 1.31)	0.022	1.13 (1.01, 1.26)	0.048	1.13 (1.01, 1.28)	0.045
Q4	1.19 (1.05, 1.34)	0.007	1.19 (1.05, 1.35)	0.006	1.21 (1.06, 1.37)	0.004
Per 1-SD increase	1.06 (1.02,1.10)	0.002	1.07 (1.02, 1.11)	0.003	1.07 (1.03,1.12)	0.001

Model 1 was unadjusted. Model 2 was adjusted for age and sex. Model 3 was further adjusted for BMI, educational level, hypertension, diabetes, annual income, smoking status, drinking status, HDL-C, TG, RBC, creatinine, and TC.

AISI, aggregate index of systemic inflammation; BMI, body mass index; CI, confidence interval; HDL-C, high-density lipoprotein cholesterol; HR, hazard ratio; NLR, neutrophil-to-lymphocyte ratio; NPAR, neutrophil percentage-to-albumin ratio; PLR, platelet-to-lymphocyte ratio; Q, quartile; RBC, red blood cell count; SII, systemic immune-inflammation index; SIRI, systemic inflammatory response index; TC, total cholesterol; TG, triglyceride.

**Figure 2:**
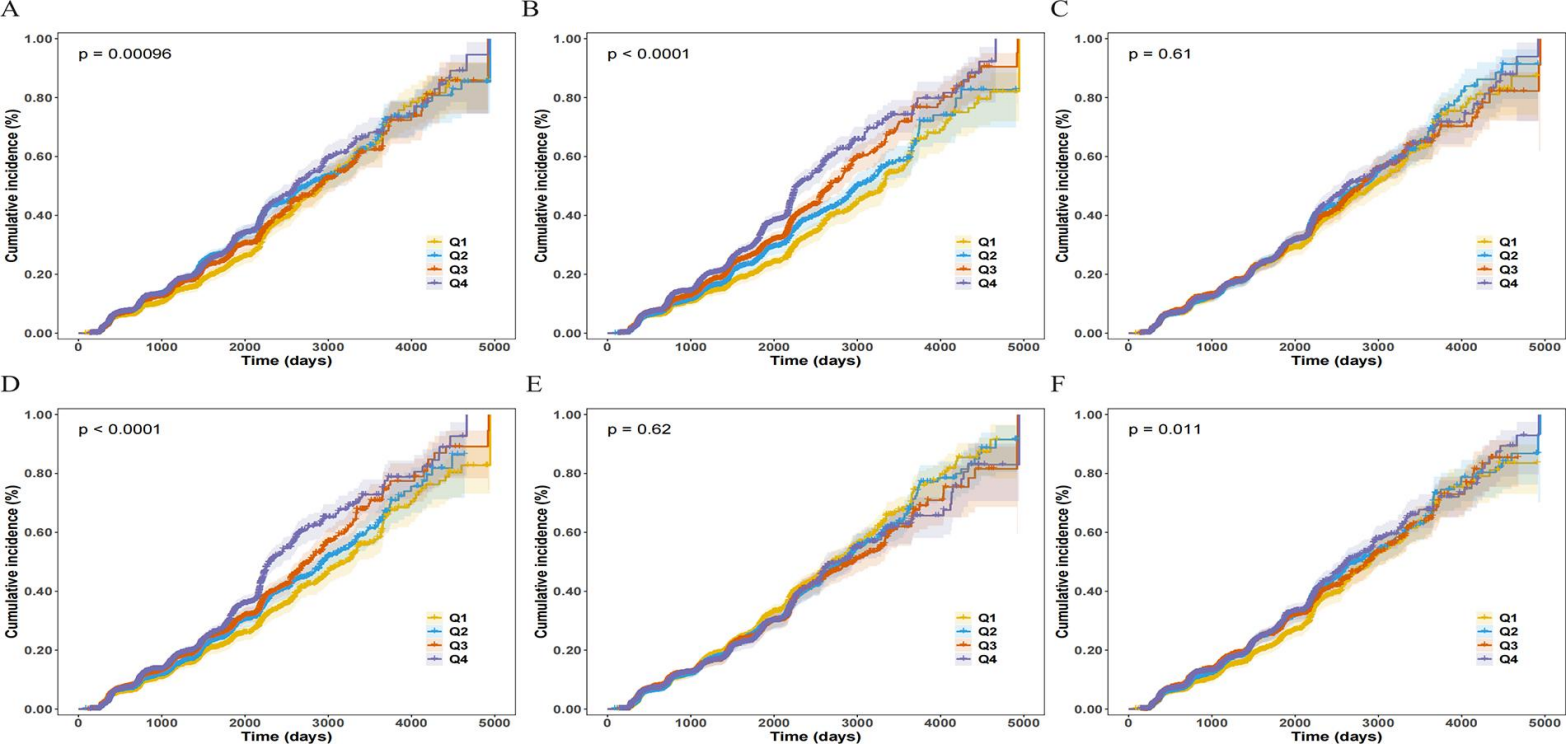
Cumulative incidence curves for incident carotid atherosclerosis (CAS) according to baseline quartiles of inflammatory indices. Panels show the Kaplan–Meier curves for **(A)** NLR, **(B)** SIRI, **(C)** SII, **(D)** AISI, **(E)** PLR, and **(F)** NPAR. The curves are stratified by quartiles (Q1–Q4), with Q1 representing the lowest quartile and Q4 the highest. Shaded areas represent the 95% confidence intervals (CIs). *P*-values were calculated using the Log-rank test.

The Kaplan–Meier survival curves (Figure 2) visually demonstrated this: in the unadjusted analysis, only the curves for SIRI, AISI, NLR, and NPAR showed significant separation across quartiles (Log-rank *P* < 0.05), whereas the curves for SII and PLR did not differ significantly (Log-rank *P* > 0.05). This suggests that the true associations of SII and PLR with CAS were masked by negative confounding factors (such as age), and their roles as independent risk factors were only revealed after adjusting for these confounders.

**Figure 3:**
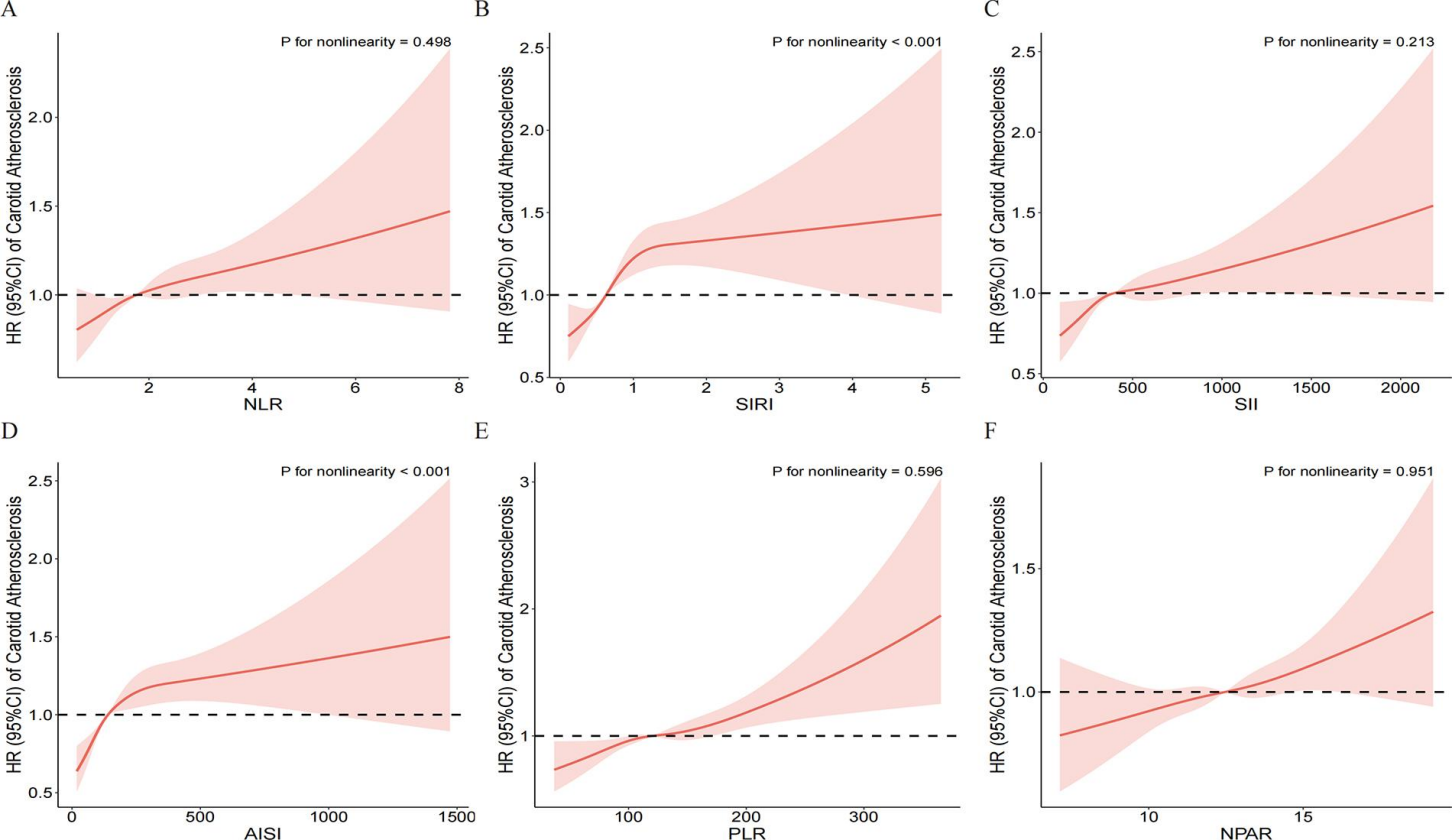
Dose-response relationship between inflammatory indices and the risk of incident carotid atherosclerosis. Restricted cubic spline models were used to visualize the associations for **(A)** NLR, **(B)** SIRI, **(C)** SII, **(D)** AISI, **(E)** PLR, and **(F)** NPAR. The solid red lines represent the estimated hazard ratios (HRs), and the pink shaded areas indicate the 95% confidence intervals (CIs). The horizontal dashed black line at HR = 1.0 represents the reference risk. The models were adjusted for gender, age, BMI, educational level, hypertension, diabetes, annual income, smoking status, drinking status, HDL-C, TG, RBC, creatinine, and TC. *P*-values for nonlinearity are shown in each panel.

Restricted cubic spline (RCS) models further explored the dose-response relationships (Figure 3). The results showed that SIRI and AISI had a significant non-linear positive association with CAS risk (*P* for non-linearity < 0.001), with the risk increasing sharply at lower levels of the indices before plateauing. In contrast, the relationships of NLR, SII, PLR, and NPAR with CAS risk were characterized by a stable linear increase (*P* for non-linearity > 0.05).

### 3.3 Comparison of predictive performance of inflammatory indices

**Figure 4:**
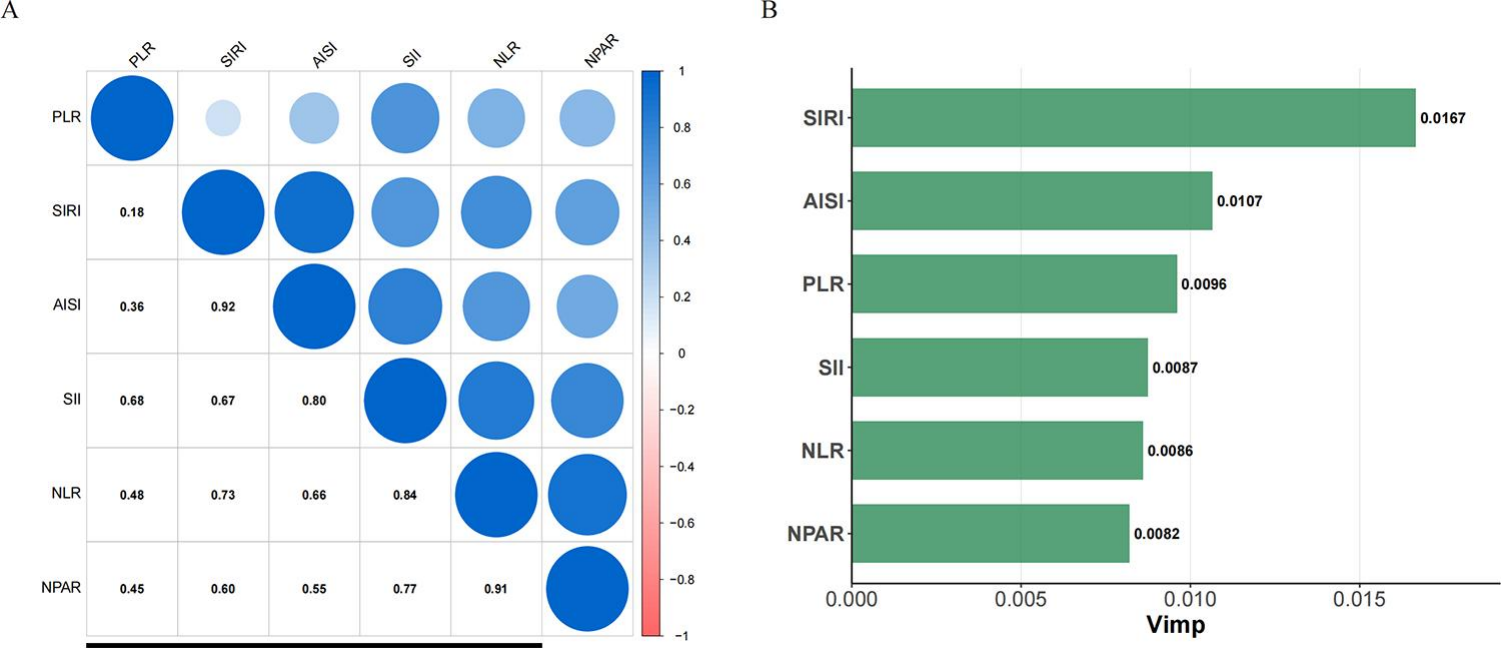
Correlation among six inflammatory indices and their predictive value for incident carotid atherosclerosis (CAS). **(A)** Spearman correlation analysis of the six baseline inflammatory indices. The size and color intensity of the circles represent the absolute value of the correlation coefficient, with blue indicating a positive correlation. The numbers in the lower triangle are the Spearman's correlation coefficients (*ρ*). **(B)** Comparison of the relative importance of the six inflammatory indices in predicting incident CAS using a Random Survival Forest (RSF) model. A higher Variable Importance (Vimp) value indicates stronger predictive power. The RSF model was adjusted for gender, age, BMI, educational level, hypertension, diabetes, annual income, smoking status, drinking status, HDL-C, TG, RBC, creatinine, and TC.

The correlation among the six inflammatory indices is presented in Figure 4A. Notably, a strong positive correlation was observed between SIRI and AISI (*ρ* = 0.92), as well as between NLR and NPAR (*ρ* = 0.91). Figure 4B reveals that, among all evaluated indices, SIRI exhibited the highest predictive value for incident CAS.

### 3.4 Subgroup and mediation analyses based on SIRI

Given that SIRI demonstrated the strongest association and predictive ability, we conducted further subgroup and mediation analyses.

Subgroup analysis (Table 2) showed that the association between SIRI and CAS risk remained stable and consistent across most prespecified subgroups, including age, BMI, smoking status, drinking status, hypertension, and diabetes, with no significant interactions detected (all *P* for interaction > 0.05). However, sex played a significant moderating role in this association (*P* for interactio*n* = 0.031). Stratified analysis revealed that the association between SIRI and CAS risk was more pronounced in males (HR: 1.15, 95% CI: 1.10–1.19) but was not statistically significant in females (HR: 1.04, 95% CI: 0.96–1.12).

**Table 2:** Subgroup analyses of the association between systemic inflammatory response index (SIRI) and risk of incident carotid atherosclerosis.

Subgroup	*N*	Adjusted HR (per 1-SD, 95% CI)	*P* value	*P* for interaction
Overall	12,289	1.12 (1.09, 1.16)	<0.001	
Age				0.145
<45	7,359	1.11 (1.05, 1.69)	<0.001	
≥45	4,930	1.14 (1.10, 1.20)	<0.001	
Gender				0.031
Female	4,810	1.04 (0.96, 1.12)	0.328	
Male	7,479	1.15 (1.10, 1.19)	<0.001	
BMI				0.435
< 24	5,878	1.11 (1.05, 1.17)	<0.001	
≥ 24	6,411	1.13 (1.09, 1.18)	<0.001	
Hypertension				0.776
No	10,420	1.13 (1.09, 1.17)	<0.001	
Yes	1,869	1.11 (1.03, 1.19)	0.004	
Diabetes				0.675
No	11,648	1.12 (1.09, 1.17)	<0.001	
Yes	641	1.10 (0.99, 1.23)	0.071	
Educational level				0.085
Low	725	1.21 (1.08,1.37)	0.001	
Medium	7,218	1.10 (1.05, 1.15)	<0.001	
High	4,346	1.15 (1.09, 1.22)	<0.001	
Annual income				0.668
Low	333	0.97 (0.68, 1.36)	0.838	
Medium	2,780	1.11 (1.04, 1.18)	<0.001	
High	9,176	1.13 (1.09, 1.18)	<0.001	
Smoking status				0.148
non-current smoker	8,752	1.08 (1.04, 1.14)	<0.001	
current smoker	3,537	1.17 (1.11, 1.24)	<0.001	
Drinking status				0.934
non-current drinker	8,904	1.12 (1.07, 1.17)	<0.001	
current drinker	3,385	1.13 (1.07–1.20)	<0.001	

Hazard Ratios (HRs) and 95% Confidence Intervals (CIs) were derived from multivariate Cox proportional hazards models.

Models were adjusted for gender, age, BMI, educational level, hypertension, diabetes, annual income, smoking status, drinking status, HDL-C, TG, RBC, and creatinine. In each subgroup analysis, the stratification variable was excluded from the adjustment model.

*P* for interaction indicates the statistical significance of the interaction between SIRI and the stratification variables.

**Figure 5:**
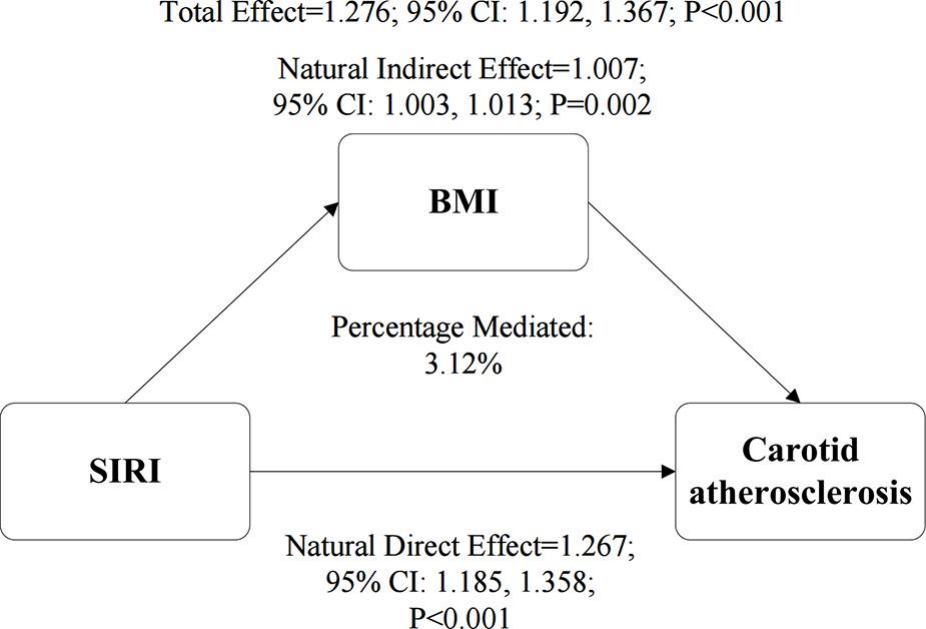
The mediating role of BMI in the association between the SIRI and CAS. The path diagram illustrates the results of the mediation analysis, showing the Natural Indirect Effect (NIE) through BMI and the Natural Direct Effect (NDE). The values represent hazard ratios with their corresponding 95% confidence intervals (CIs), which were derived from a bootstrap procedure with 1,000 resamples. The proportion of the total effect mediated by BMI is also presented. All analyses were adjusted for gender, age, educational level, hypertension, diabetes, annual income, smoking status, drinking status, HDL-C, TG, RBC, creatinine, and TC.

After confirming significant positive associations between SIRI and BMI (Supplementary Table S6) and between BMI and CAS (Supplementary Table S7), we performed a mediation analysis to investigate the role of BMI in the SIRI-CAS pathway. The results (Figure 5) indicated that BMI partially mediated this association. The natural indirect effect (NIE) mediated by BMI was statistically significant (HR = 1.007, *P* = 0.002), explaining 3.12% of the total effect of SIRI. Concurrently, the natural direct effect (NDE) was also significant (NDE = 1.267, *P* < 0.001), indicating that SIRI's effect on CAS risk is predominantly direct or mediated through other non-BMI pathways.

### 3.5 Sensitivity analysis

To test the robustness of the results and assess potential reverse causality bias, a sensitivity analysis was conducted by excluding participants diagnosed with CAS within the first year of follow-up, and re-analyzing the remaining cohort of 11,903 individuals. In the refitted Cox models, the associations of NLR, SIRI, SII, AISI, PLR, and NPAR with CAS risk remained robust and statistically significant (Supplementary Table S8). Crucially, the random survival forest model constructed in this sensitivity analysis cohort reconfirmed that SIRI was still the strongest inflammatory predictor of CAS risk (Supplementary Figure S1). Furthermore, to assess the potential impact of unmeasured confounding, we calculated the E-value for the primary finding. The E-value for the association between SIRI and incident CAS was 1.77. This indicates that an unmeasured confounder would need to be associated with both SIRI and the outcome by a risk ratio of at least 1.77 to explain away the observed association, suggesting that the findings are reasonably robust to potential unmeasured confounding. This series of results strongly demonstrates the robustness of our main findings.

## 4 Discussion

In this large-scale prospective cohort study, we systematically evaluated the associations between six composite inflammatory indices, derived from routine blood tests, and the risk of incident CAS. Our principal finding reveals that all evaluated inflammatory indices were significantly and independently associated with incident CAS. Among them, the SIRI emerged as the marker with the strongest and most robust association with CAS risk. This discovery offers novel insights into the role of systemic inflammation during the early stages of atherosclerosis. Our findings lend strong support to the established paradigm that inflammation is a core driver throughout the entire process of atherosclerosis, from initiation to progression.

By integrating the counts of three key immune cells—neutrophils, monocytes, and lymphocytes—SIRI effectively captures the paradigm shift in our understanding of atherogenesis from “lipids to leukocytes” (18). While other indices like NLR or PLR capture only facets of this response, SIRI provides a more multidimensional depiction of the body's immuno-inflammatory status. Mechanistically, this association is underpinned by the complex interplay between innate and adaptive immunity (19, 20). The numerator of SIRI (neutrophils and monocytes) represents the rapid innate immune arm. Upon activation by pattern-recognition receptors, these cells not only release reactive oxygen species and proteases but also activate the NLRP3 inflammasome. Recent evidence highlights the NLRP3 inflammasome as a critical signal transducer that orchestrates endothelial injury, promotes foam cell formation, and induces pyroptosis, thereby accelerating plaque progression (21). Furthermore, monocytes differentiate into macrophages, which are pivotal in inducing plaque vulnerability. These macrophages accumulate in the lipid-rich necrotic core, driving inflammation and matrix degradation, which are hallmarks of high-risk plaques (22). Conversely, the denominator (lymphocytes) reflects the adaptive immune response. A lower lymphocyte count often indicates a depletion of protective regulatory T-cells (Tregs), leading to a failure in immunomodulatory checks that would otherwise inhibit plaque growth (20). Crucially, systemic inflammation drives the phenotypic transformation of plaques towards vulnerability. Elevated inflammatory markers are intrinsically linked to macroscopic high-risk features such as microcalcification, neovascularization, and lipid-rich necrotic cores. This “thromboinflammation” cascade can degrade the extracellular matrix and thin the fibrous cap, increasing the risk of rupture even in non-stenotic plaques (23, 24). Thus, an elevated SIRI does not merely reflect a blood count anomaly but signals a comprehensive “inflammatory plaque phenotype” prone to instability.

This pathophysiological link is not only evident at the cellular level but is also corroborated by genetic studies. For instance, as demonstrated in a study on community and high-risk stroke populations in Southwest China, genetic polymorphisms related to inflammatory responses and endothelial function are also closely associated with susceptibility to carotid atherosclerosis (25, 26). This adds a layer of genetic evidence to the fundamental role of inflammation in atherosclerosis. Therefore, an elevated SIRI, reflecting a pro-inflammatory imbalance characterized by increased neutrophils/monocytes and decreased lymphocytes, is grounded in a solid pathophysiological basis as a strong risk indicator for CAS.

Our findings are highly consistent with previous studies linking SIRI to clinical endpoints such as coronary heart disease, stroke, and cardiovascular mortality (27–30), and extend these findings to the subclinical stage of atherosclerosis—incident CAS—thereby reinforcing the potential value of these indices in the primary prevention of cardiovascular disease.

A noteworthy observation was that while the associations of SII and PLR with CAS were not significant in unadjusted analyses, all six inflammatory indices demonstrated independent statistical significance after rigorous adjustment for a wide array of traditional cardiovascular risk factors, including age, sex, blood pressure, lipids, glucose, and smoking status. This underscores the critical importance of multivariate adjustment to eliminate confounding effects when evaluating these novel biomarkers, suggesting that even seemingly weaker inflammatory signals may play a non-negligible role within a complex risk network. Furthermore, our RSF model confirmed that, after adjusting for numerous covariates, SIRI exhibited the highest variable importance among the six indices. Its ranking consistently remained at the top, a result validated in sensitivity analyses, attesting to the robustness of our findings.

Regarding the sex-stratified analysis, we identified a significant interaction between sex and SIRI on the risk of CAS (*P* for interaction = 0.031), with a prominent association observed in men but not in women. However, these sex-specific results warrant cautious interpretation. As noted, the interaction *p*-value was borderline, and the smaller number of events among women may have reduced the statistical power to detect an association. Although the female subgroup had a substantial sample size (*n* = 4,810), the number of incident events was significantly lower compared to men (709 vs. 1,723 events). This discrepancy suggests that the non-significant finding in women may partly reflect limited statistical power rather than a definitive absence of risk. While biological factors—such as the potential protective effects of estrogen on endothelial function (31) or sex-specific differences in plaque stability (32–34)—might contribute to a differential risk profile, current evidence precludes a definitive mechanistic conclusion. Therefore, we emphasize that the observed sex disparity may be influenced by statistical limitations, and the potential for SIRI to predict risk in women should not be prematurely dismissed; future studies with larger event rates are needed for confirmation.

Similarly, regarding the diabetes subgroup, although the association between SIRI and CAS showed a borderline significance (*P* = 0.071), this should be interpreted in the context of statistical power. The sample size of the diabetes subgroup was relatively small (*n* = 641), which likely limited the power to detect a statistically significant effect. Importantly, the hazard ratio in the diabetes subgroup (1.10) was highly comparable to that of the non-diabetes subgroup (1.12), and the interaction test was non-significant (*P* for interaction = 0.675). These results suggest that the predictive value of SIRI remains consistent across glycemic statuses, and the lack of significance in the diabetes subgroup is more likely attributable to the small sample size rather than a true absence of biological association.

From an epidemiological perspective, a large cross-sectional study based on the NHANES database has confirmed a close link between SIRI and obesity. Another study also observed a significant association between SIRI and obesity in sedentary adults (35, 36). At the molecular level, chronic inflammation is a key driver in the development of obesity; for example, the NLRP3 inflammasome has been shown to play a causal role in inducing metabolic disorders and adipose tissue expansion (37). Consequently, it is plausible that an elevated SIRI, as a marker of systemic inflammation, would predict an increase in BMI. Obesity is widely recognized as an independent risk factor for carotid atherosclerosis. This is supported by multiple studies: one prospective study showed that the metabolic heterogeneity of obesity directly influences CAS risk, while another observed a strong association between obesity and CAS in patients with type 1 diabetes (38, 39). The persistent systemic inflammation, as indicated by an elevated SIRI, may ultimately increase CAS risk by promoting the development and progression of obesity (reflected by a higher BMI). This finding not only uncovers a potential pathophysiological axis of “inflammation-obesity-atherosclerosis” but also offers important clinical implications: combining anti-inflammatory therapies with weight management may represent a more synergistic strategy for the prevention and intervention of CAS.

Crucially, our results have direct implications for clinical practice, addressing the urgent need for cost-effective screening tools in resource-constrained settings. The versatility of SIRI as a scalable risk stratification tool has been increasingly recognized across a spectrum of diseases. Recent studies have validated its prognostic value in acute ischemic stroke (40), ST-segment elevation myocardial infarction (41), and metabolic complications such as diabetic retinopathy (42) and metabolic dysfunction-associated fatty liver disease (MAFLD) (43). Furthermore, a recent study highlighted that elevated SIRI is associated with lower bone mineral density and increased fracture risk in elderly hypertensive patients (44). This collective evidence suggests that SIRI captures a “systemic inflammatory phenotype” that simultaneously compromises vascular, metabolic, and skeletal health, making it particularly valuable for managing elderly populations with multiple comorbidities. While classical inflammatory biomarkers, particularly high-sensitivity C-reactive protein (hs-CRP), are established predictors of cardiovascular risk, cell-based indices like SIRI may offer distinct and complementary biological information. hs-CRP is a downstream acute-phase protein produced by the liver in response to cytokine stimulation (e.g., IL-6). In contrast, SIRI directly reflects the balance of the cellular effectors of inflammation (neutrophils and monocytes) and the adaptive immune response (lymphocytes). Previous research suggests that these cellular indices may arguably be more sensitive in specific atherosclerotic contexts. For instance, a cross-sectional study by Park et al. demonstrated that while cell-based indices (such as NLR and PLR) were significant predictors of severe carotid plaque burden, hs-CRP failed to show a significant association in the same cohort (45). This discrepancy implies that cell count fluctuations might precede or occur independently of the systemic protein surge in early-stage atherosclerosis or specific plaque phenotypes. Therefore, SIRI should not be viewed merely as a surrogate for hs-CRP, but potentially as a marker capturing a different dimension of the immuno-inflammatory landscape—specifically, the cellular “readiness” for atherogenesis.

Consistent with these findings, we propose that SIRI can serve as a cost-effective tool for initial risk stratification. Unlike high-sensitivity C-reactive protein (hs-CRP) or interleukin-6, which require specific assays and additional costs, SIRI can be instantly calculated from a standard complete blood count (CBC)—a routine test performed in almost all clinical encounters. In terms of practical implementation, we propose a two-step screening strategy. First, SIRI could be integrated into Electronic Health Record (EHR) systems to automatically calculate and flag patients with elevated values during routine visits. This automation would allow for large-scale risk assessment without increasing the clinician's workload. Second, for individuals identified with a high SIRI (particularly men or those with traditional intermediate risk), clinicians should consider a more aggressive diagnostic workup, such as referral for carotid ultrasound to detect subclinical plaque. This approach transforms SIRI from a mere statistical marker into an actionable clinical tool, enabling the allocation of more expensive imaging resources to the patients who need them most.

The strengths of our study are multifold. First, the prospective design and large sample size allowed us to establish temporal causality and achieve high statistical power. Second, we employed a variety of robust statistical methods, including multivariate Cox regression, RCS, Random Survival Forest, and mediation analysis, for a comprehensive and in-depth analysis. Third, we performed meticulous adjustment for a large number of potential confounders. We also calculated the E-value to quantify the potential impact of unmeasured confounding and assessed the possibility of reverse causality through sensitivity analyses (e.g., excluding individuals who developed CAS within the first year of follow-up), which enhances the reliability of our results.

Nevertheless, several limitations should be acknowledged. First, despite meticulous adjustment for multiple confounders, the possibility of residual confounding from unmeasured factors cannot be entirely ruled out. Specifically, data on genetic predisposition, psychological stress, physical activity, sleep quality, and detailed medication history (e.g., the use of statins or non-steroidal anti-inflammatory drugs) were not fully available for adjustment. Additionally, precise details on dietary patterns (e.g., Western diet) were not captured. However, we calculated the E-value to assess the robustness of our findings. The E-value for the association between SIRI and CAS was 1.77, suggesting that an unmeasured confounder would need to be associated with both the exposure and the outcome by a risk ratio of at least 1.77 to explain away the observed association. This indicates that our results are moderately robust. Second, a limitation of our study is the absence of data on classical inflammatory biomarkers, such as hs-CRP or IL-6. This precluded a direct statistical comparison (e.g., C-statistic comparison or net reclassification improvement) to determine whether SIRI adds incremental predictive value beyond these established markers. However, as noted above, the primary clinical value of SIRI lies in its accessibility. Since it is derived from routine CBC without additional cost, it remains a valuable tool for initial risk stratification in settings where hs-CRP is not routinely measured. Third, the inflammatory indices were derived from a single baseline measurement. We acknowledge that such a “snapshot” may not fully capture the long-term inflammatory burden or cumulative exposure, as inflammation levels can fluctuate due to transient factors (e.g., minor infections or physiological stress). This intra-individual variability is known to introduce random measurement error, leading to regression dilution bias. Consequently, this bias tends to attenuate the association towards the null, suggesting that the hazard ratios reported in our study are likely conservative estimates, and the true strength of the relationship between SIRI and CAS risk could be even greater than observed. Future studies incorporating repeated measurements are warranted to more accurately evaluate the cumulative exposure and long-term patterns of multiple inflammatory indices on atherosclerosis. Fourth, the diagnosis of CAS relied on carotid ultrasound. While ultrasound is the standard first-line tool for large-scale population screening due to its non-invasiveness and cost-effectiveness, it has inherent limitations compared to advanced imaging techniques such as intravascular ultrasound (IVUS) or CT angiography (CTA). Specifically, ultrasound is operator-dependent, and inter-observer variability may exist. Furthermore, our outcome was defined as a composite of both CIMT thickening (≥ 1.0 mm) and plaque formation (≥ 1.5 mm). While this definition increases sensitivity, it limits our ability to distinguish between early vascular remodeling and established atherosclerosis, and the lower resolution of ultrasound may miss small or early-stage plaques compared to IVUS. However, such non-differential misclassification generally biases the results towards the null, leading to an underestimation of the true association strength. Therefore, despite this limitation, the significant positive association observed in our study remains noteworthy. fifth, the median follow-up period was 2.15 years, which is relatively short for a chronic disease such as atherosclerosis. This duration limits our ability to draw definitive conclusions regarding long-term risk prediction and the very early initiation phase of the disease. Although a significant association was found, our findings primarily reflect short-to-medium-term risk. A longer follow-up duration is necessary to confirm whether SIRI predicts long-term atherosclerotic trajectories and to fully elucidate its role in disease initiation. Finally, our study population consisted exclusively of Chinese adults. While this provides valuable insights for this specific demographic, differences in genetic backgrounds, dietary habits, and environmental exposures may limit the generalizability of our findings to other racial or ethnic groups. Therefore, caution should be exercised when extrapolating these results to non-Chinese populations.

In conclusion, our study is the first to demonstrate in a large health screening cohort that SIRI is a robust and independent predictor of incident CAS, particularly in the short-to-medium term, and its utility for risk identification consistently outperforms other composite inflammatory indices, particularly in men. Given that SIRI can be easily and inexpensively calculated from a routine complete blood count, it has the potential to become a highly cost-effective risk stratification tool for the early identification of individuals at high risk for CAS in clinical practice. Future research should focus on: 1) validating our findings in multi-ethnic cohorts and diverse populations to confirm their generalizability; 2) exploring the value of serial SIRI measurements for dynamic risk assessment; and 3) ultimately, determining through randomized controlled trials whether targeted reduction of systemic inflammation (e.g., through lifestyle modifications, statins, or novel anti-inflammatory drugs) can effectively lower the atherosclerotic risk indicated by a high SIRI.

## 5 Conclusion

In this large-scale cohort, we provide the first evidence that SIRI is a superior and independent predictor of incident CAS over a short-to-medium term follow-up, outperforming other composite inflammatory indices. Our findings highlight a pivotal “inflammation-obesity-atherosclerosis” axis, suggesting that systemic inflammation is closely linked to the risk of early-stage atherosclerosis, partially through metabolic dysregulation. Given that SIRI is derived from routine blood counts, it represents an accessible and cost-effective tool for early risk stratification in clinical practice. Integrating SIRI into screening protocols could significantly enhance the primary prevention of cardiovascular disease by identifying high-risk individuals before structural vascular changes become advanced. Future studies are warranted to validate these findings in diverse populations and to evaluate the prognostic value of longitudinal SIRI trajectories in capturing dynamic inflammatory status.

## Data Availability

The original contributions presented in this study are included in the article. Further inquiries can be directed to the corresponding author.
