# Supplementary material for: Comparative value of novel inflammatory indices in predicting incident carotid atherosclerosis: SIRI outperforms other markers in a general population
Source: Front Cardiovasc Med. 2026 Feb 18;13:1760659. doi: 10.3389/fcvm.2026.1760659 (PMC12957181; doi:10.3389/fcvm.2026.1760659)
Supplement: Supplementary file 1 [file Datasheet1.docx]

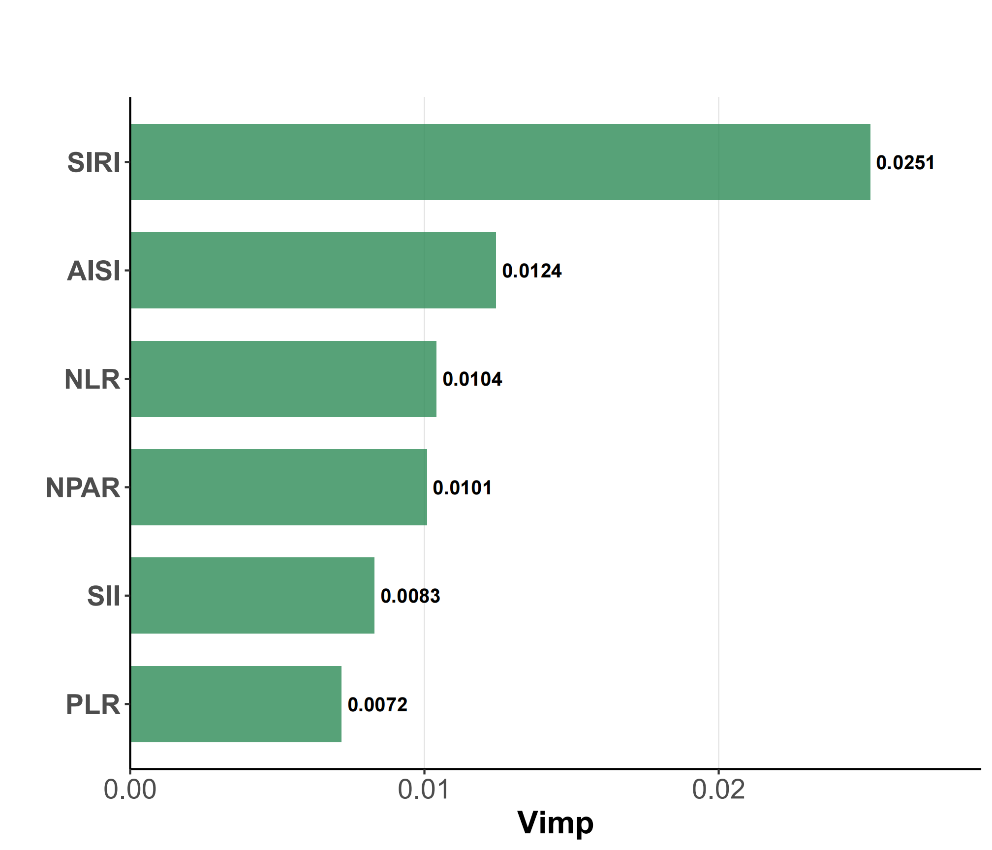


**Supplementary Figure S1.** Variable importance of six systemic inflammatory indices in the prediction of carotid atherosclerosis using a Random Forest model.

**Supplementary Table S1.** Comparison of baseline characteristics between participants included in the final analysis and those excluded due to lack of carotid ultrasound examination.

| **Characteristic** | **Included Participants**  **(N = 12,289)** | **Excluded Participants**  **(N = 89,563)** |
| --- | --- | --- |
| **Age (years)** | 42 (37, 48) | 41 (34, 49) |
| **BMI (kg/m²)** | 24.2 (22.0, 26.5) | 23.7 (21.3, 26.2) |
| **Gender, n (%)** |  |  |
| Female | 4,810 (39.1%) | 43,894 (49.0%) |
| Male | 7,479 (60.9%) | 45,669 (51.0%) |
| **Hypertension, n (%)** |  |  |
| No | 10,420 (84.8%) | 77,357 (86.4%) |
| Yes | 1,869 (15.2%) | 12,206 (13.6%) |
| **Diabetes, n (%)** |  |  |
| No | 11,648 (94.8%) | 84,637 (94.5%) |
| Yes | 641 (5.2%) | 4,926 (5.5%) |
| **Educational level, n (%)** |  |  |
| Low | 725 (5.9%) | 6,643 (7.4%) |
| Medium | 7,218 (58.7%) | 59,196 (66.1%) |
| High | 4,346 (35.4%) | 23,724 (26.5%) |
| **Annual income, n (%)** |  |  |
| Low | 333 (2.7%) | 2,927 (3.3%) |
| Medium | 2,780 (22.6%) | 35,689 (39.8%) |
| High | 9,176 (74.7%) | 50,947 (56.9%) |
| **Smoking status, n (%)** |  |  |
| non-current smoker | 8,752 (71.2%) | 65,782 (73.4%) |
| current smoker | 3,537 (28.8%) | 23,781 (26.6%) |
| **Drinking status, n (%)** |  |  |
| non-current drinker | 8,904 (72.5%) | 70,853 (79.1%) |
| current drinker | 3,385 (27.5%) | 18,710 (20.9%) |
| **TG (mmol/L)** | 1.17 (0.82, 1.73) | 1.06 (0.74, 1.59) |
| **HDL-C (mmol/L)** | 1.31 (1.10, 1.59) | 1.35 (1.12, 1.63) |
| **TC (mmol/L)** | 4.70 (4.19, 5.30) | 4.60 (4.00, 5.13) |
| **Note:** Values are presented as n (%) for categorical variables and median (interquartile range, Q1–Q3) for continuous variables.  **Abbreviations:** BMI, body mass index; TG, triglyceride; HDL-C, high-density lipoprotein cholesterol; TC, total cholesterol. | | |

**Supplementary Table S2.** Comparison of baseline characteristics between the final study population and participants excluded due to missing inflammatory biomarkers.

| **Characteristic** | **Included Participants**  **(N = 12,289)** | **Excluded Participants**  **N = 38,390** |
| --- | --- | --- |
| **Age (years)** | 42 (37, 48) | 43 (38, 52) |
| **BMI (kg/m²)** | 24.2 (22.0, 26.5) | 24.3 (22.1, 26.6) |
| **Gender, n (%)** |  |  |
| Female | 4,810 (39.1%) | 14,844 (38.7%) |
| Male | 7,479 (60.9%) | 23,546 (61.3%) |
| **Hypertension, n (%)** |  |  |
| No | 10,420 (84.8%) | 32,747 (85.3%) |
| Yes | 1,869 (15.2%) | 5,643 (14.7%) |
| **Diabetes, n (%)** |  |  |
| No | 11,648 (94.8%) | 35,779 (93.2%) |
| Yes | 641 (5.2%) | 2,611 (6.8%) |
| **Educational level, n (%)** |  |  |
| Low | 725 (5.9%) | 3,420 (8.9%) |
| Medium | 7,218 (58.7%) | 22,892 (59.6%) |
| High | 4,346 (35.4%) | 12,078 (31.5%) |
| **Annual income, n (%)** |  |  |
| Low | 333 (2.7%) | 1,264 (3.3%) |
| Medium | 2,780 (22.6%) | 9,804 (25.5%) |
| High | 9,176 (74.7%) | 27,322 (71.2%) |
| **Smoking status, n (%)** |  |  |
| non-current smoker | 8,752 (71.2%) | 27,695 (72.1%) |
| current smoker | 3,537 (28.8%) | 10,695 (27.9%) |
| **Drinking status, n (%)** |  |  |
| non-current drinker | 8,904 (72.5%) | 27,656 (72.0%) |
| current drinker | 3,385 (27.5%) | 10,734 (28.0%) |
| **TG (mmol/L)** | 1.17 (0.82, 1.73) | 1.22 (0.85, 1.77) |
| **HDL-C (mmol/L)** | 1.31 (1.10, 1.59) | 1.32 (1.10, 1.60) |
| **TC (mmol/L)** | 4.70 (4.19, 5.30) | 4.70 (4.20, 5.30) |
| **Note:** Values are presented as n (%) for categorical variables and median (interquartile range, Q1–Q3) for continuous variables.  **Abbreviations:** BMI, body mass index; TG, triglyceride; HDL-C, high-density lipoprotein cholesterol; TC, total cholesterol. | | |

**Supplementary Table S3.** Demographic and clinic characteristics.

| **Characteristic** | **Carotid atherosclerosis** | | | **P value** |
| --- | --- | --- | --- | --- |
|  | **Overall  N = 12,289** | **No  N = 9,857** | **Yes  N = 2,432** |  |
|  |  |  |  |  |
| **Age (years)** | 42 (37, 48) | 41 (36, 47) | 48 (43, 54) | <0.001^1^ |
| **BMI** **(kg/m²)** | 24.2 (22.0, 26.5) | 24.0 (21.8, 26.4) | 24.8 (22.9, 27.0) | <0.001^1^ |
| **Gender, n (%)** |  |  |  | <0.001^2^ |
| Female | 4,810 (39.1%) | 4,101 (41.6%) | 709 (29.2%) |  |
| Male | 7,479 (60.9%) | 5,756 (58.4%) | 1,723 (70.8%) |  |
| **Hypertension, n (%)** |  |  |  | <0.001^2^ |
| No | 10,420 (84.8%) | 8,590 (87.1%) | 1,830 (75.2%) |  |
| Yes | 1,869 (15.2%) | 1,267 (12.9%) | 602 (24.8%) |  |
| **Diabetes, n (%)** |  |  |  | <0.001^2^ |
| No | 11,648 (94.8%) | 9,467 (96.0%) | 2,181 (89.7%) |  |
| Yes | 641 (5.2%) | 390 (4.0%) | 251 (10.3%) |  |
| **Educational level, n (%)** |  |  |  | <0.001^2^ |
| Low | 725 (5.9%) | 506 (5.2%) | 219 (9.0%) |  |
| Medium | 7,218 (58.7%) | 5,713 (57.9%) | 1,505 (61.9%) |  |
| High | 4,346 (35.4%) | 3,638 (36.9%) | 708 (29.1%) |  |
| **Annual income, n (%)** |  |  |  | <0.001^2^ |
| Low | 333 (2.7%) | 283 (2.9%) | 50 (2.1%) |  |
| Medium | 2,780 (22.6%) | 2,085 (21.1%) | 695 (28.6%) |  |
| High | 9,176 (74.7%) | 7,489 (76.0%) | 1,687 (69.4%) |  |
| **Smoking status, n (%)** |  |  |  | <0.001^2^ |
| non-current smoker | 8,752 (71.2%) | 7,197 (73.0%) | 1,555 (63.9%) |  |
| current smoker | 3,537 (28.8%) | 2,660 (27.0%) | 877 (36.1%) |  |
| **Drinking status, n (%)** |  |  |  | <0.001^2^ |
| non-current drinker | 8,904 (72.5%) | 7,262 (73.7%) | 1,642 (67.5%) |  |
| current drinker | 3,385 (27.5%) | 2,595 (26.3%) | 790 (32.5%) |  |
| **TG** **(mmol/L)** | 1.17 (0.82, 1.73) | 1.13 (0.79, 1.69) | 1.36 (0.96, 1.91) | <0.001^1^ |
| **HDL-C** **(mmol/L)** | 1.31 (1.10, 1.59) | 1.33 (1.11, 1.60) | 1.26 (1.06, 1.53) | <0.001^1^ |
| **TC** **(mmol/L)** | 4.70 (4.19, 5.30) | 4.60 (4.10, 5.20) | 4.90 (4.40, 5.50) | <0.001^1^ |
| **RBC (10^12^/L)** | 4.87 (4.51, 5.19) | 4.86 (4.50, 5.18) | 4.91 (4.59, 5.20) | <0.001^1^ |
| **Creatinine μmol/L** | 73 (61, 83) | 72 (61, 83) | 75 (65, 85) | <0.001^1^ |
| **Lymphocyte count (10^9^/L)** | 1.86 (1.55, 2.23) | 1.86 (1.55, 2.22) | 1.90 (1.58, 2.29) | <0.001^1^ |
| **Monocyte count (10^9^/L)** | 0.36 (0.28, 0.45) | 0.35 (0.28, 0.45) | 0.38 (0.29, 0.48) | <0.001^1^ |
| **Neutrophil count (10^9^/L)** | 3.31 (2.70, 4.05) | 3.29 (2.68, 4.03) | 3.38 (2.77, 4.16) | <0.001^1^ |
| **Neutrophil percentage (%)** | 58 (53, 63) | 58 (53, 63) | 58 (53, 63) | 0.320^1^ |
| **ALB** **g/L** | 4.70 (4.50, 4.80) | 4.70 (4.50, 4.80) | 4.60 (4.50, 4.80) | <0.001^1^ |
| **NLR** | 1.76 (1.40, 2.23) | 1.76 (1.40, 2.23) | 1.76 (1.41, 2.25) | 0.422^1^ |
| **SIRI** | 0.63 (0.44, 0.89) | 0.62 (0.44, 0.88) | 0.67 (0.46, 0.96) | <0.001^1^ |
| **SII** | 395 (297, 529) | 397 (298, 533) | 389 (295, 515) | 0.032^1^ |
| **AISI** | 140 (94, 211) | 139 (93, 208) | 146 (98, 219) | <0.001^1^ |
| **PLR** | 120 (98, 149) | 122 (99, 150) | 115 (94, 142) | <0.001^1^ |
| **NPAR** | 12.47 (11.24, 13.70) | 12.47 (11.23, 13.71) | 12.48 (11.29, 13.70) | 0.683^1^ |
| ^1^Wilcoxon rank sum test  ^2^Pearson's Chi-squared test  **Note:** Values are presented as n (%) for categorical variables and median (interquartile range, Q1–Q3) for continuous variables.  Abbreviations: BMI, body mass index; TG, triglyceride; HDL-C, high-density lipoprotein cholesterol; TC, total cholesterol; RBC, red blood cell; SIRI, system inflammation response index; SII, systemic immune-inflammation index; AISI, aggregate index of systemic inflammation; PLR, platelet-to-lymphocyte ratio; NPAR, neutrophil percentage-to-albumin ratio. | | | | |

**Supplementary Table S4.** Test of Proportional Hazards Assumption Based on Scaled Schoenfeld Residuals.

| **Variable** | **PH assumption test** |
| --- | --- |
| SIRI | 0.152 |
| SII | 0.641 |
| AISI | 0.187 |
| NLR | 0.588 |
| PLR | 0.193 |
| NPAR | 0.236 |
| The proportional hazards assumption was tested using scaled Schoenfeld residuals. A P-value > 0.05 indicates that the assumption is not violated. | |

**Supplementary Table S5.** Collinearity Diagnostics (Variance Inflation Factors) for Covariates in the Fully Adjusted Model.

| **Variable** | **VIF** |
| --- | --- |
| **Demographic characteristics** |  |
| Age | 1.23 |
| Sex | 3.12 |
| **Socioeconomic status** |  |
| Educational level | 1.24 |
| Annual income | 1.17 |
| **Lifestyle factors** |  |
| Smoking status | 1.23 |
| drinking status | 1.26 |
| **Anthropometric and Clinical characteristics** |  |
| BMI | 1.50 |
| Hypertension | 1.18 |
| Diabetes | 1.07 |
| TC | 1.32 |
| TG | 1.63 |
| HDL-C | 1.93 |
| creatinine | 1.83 |
| RBC | 1.93 |
| VIF < 5 indicates no severe multicollinearity. |  |

**Supplementary Table S6.** Independent association of SIRI with BMI.

|  | **Beta (95% CI)** | **p-value** |
| --- | --- | --- |
| SIRI |  |  |
| Per 1-SD increase | 0.12 (0.07, 0.17) | <0.001 |
| Beta coefficients were derived from a multivariate regression model, with BMI as the dependent variable. The model was adjusted for gender, age, educational level, hypertension, diabetes, annual income, smoking status, drinking status, HDL-C, TG, RBC, creatinine, and TC. | | |

**Supplementary Table S7.** Analysis of BMI as an independent risk factor for carotid atherosclerosis.

|  | **HR (95% CI)** | **p-value** |
| --- | --- | --- |
| BMI |  |  |
| Per 1-SD increase | 1.11 (1.05, 1.17) | <0.001 |
| Hazard Ratios (HRs) were derived from a multivariate Cox proportional hazards model, adjusted for gender, age, SIRI, educational level, hypertension, diabetes, annual income, smoking status, drinking status, HDL-C, TG, RBC, creatinine, and TC. | | |

**Supplementary Table S8.** Sensitivity analysis to address potential reverse causation: associations of systemic inflammatory indices with risk of incident carotid atherosclerosis by excluding cases diagnosed in the first year of follow-up.

|  | **Model 1** |  | **Model 2** |  | **Model 3** |  |
| --- | --- | --- | --- | --- | --- | --- |
|  | **HR (95%CI)** | **p-value** | **HR (95% CI)** | **p-value** | **HR (95% CI)** | **p-value** |
| **NLR** |  |  |  |  |  |  |
| Q1 | — |  | — |  | — |  |
| Q2 | 1.23 (1.09, 1.39) | <0.001 | 1.22 (1.08,1.38) | 0.001 | 1.21 (1.07, 1.36) | 0.003 |
| Q3 | 1.11 (0.98, 1.26) | 0.095 | 1.16 (1.02, 1.31) | 0.021 | 1.14 (1.01, 1.30) | 0.037 |
| Q4 | 1.25 (1.10, 1.41) | <0.001 | 1.30 (1.15, 1.47) | <0.001 | 1.30 (1.15, 1.47) | <0.001 |
| **Per 1-SD increase** | 1.05 (1.01, 1.10) | 0.011 | 1.07 (1.03, 1.11) | 0.001 | 1.07 (1.03, 1.12) | <0.001 |
| **SIRI**  Q1  Q2  Q3  Q4  **Per 1-SD increase**  **SII**  Q1  Q2  Q3  Q4  **Per 1-SD increase**  **AISI**  Q1  Q2  Q3  Q4  **Per 1-SD increase**  **PLR**  Q1  Q2  Q3  Q4  **Per 1-SD increase**  **NPAR**  Q1  Q2  Q3  Q4  **Per 1-SD increase** | —  1.20 (1.05, 1.37)  1.42 (1.25, 1.61)  1.76 (1.55, 1.99)  1.14 (1.10, 1.17)  —  1.06 (0.94, 1.20)  1.05 (0.93, 1.19)  1.08 (0.94, 1.21)  1.02 (0.98, 1.06)  —  1.16 (1.02, 1.32)  1.30 (1.15, 1.48)  1.56 (1.38, 1.77)  1.11 (1.07, 1.15)  —  0.91 (0.81, 1.03)  0.92 (0.81, 1.03)  0.85 (0.75, 0.96)  0.96 (0.91, 1.00)  —  1.22 (1.08, 1.38)  1.15 (1.01, 1.30)  1.20 (1.06, 1.36)  1.05 (1.01,1.10) | 0.006  <0.001  <0.001  <0.001  0.313  0.403  0.294  0.438  0.020  <0.001  <0.001  <0.001  0.122  0.157  0.011  0.057  0.002  0.029  0.004  0.018 | —  1.19 (1.04, 1.36)  1.42 (1.25, 1.61)  1.75 (1.54, 1.98)  1.15 (1.11, 1.19)  —  1.17 (1.04, 1.32)  1.17 (1.03, 1.32)  1.28 (1.13, 1.45)  1.08 (1.03, 1.12)  —  1.19 (1.05, 1.35)  1.45 (1.28, 1.64)  1.70 (1.50, 1.92)  1.14 (1.10, 1.18)  —  1.04 (0.92, 1.17)  1.06 (0.94, 1.20)  1.06 (0.94, 1.21)  1.03 (0.99, 1.08)  —  1.14 (1.01, 1.29)  1.11 (0.98, 1.26)  1.21 (1.07, 1.37)  1.06 (1.01, 1.11) | 0.010  <0.001  <0.001  <0.001  0.009  0.012  <0.001  <0.001  0.007  <0.001  <0.001  <0.001  0.565  0.357  0.342  0.159  0.034  0.092  0.003  0.014 | —  1.17 (1.02, 1.33)  1.39 (1.22, 1.58)  1.69 (1.49, 1.92)  1.15 (1.11, 1.19)  —  1.15 (1.02, 1.29)  1.14 (1.01, 1.29)  1.23 (1.09, 1.40)  1.07 (1.02, 1.11)  —  1.17 (1.03, 1.33)  1.39 (1.22, 1.58)  1.60 (1.41, 1.81)  1.13 (1.09, 1.17)  —  1.06 (0.94, 1.20)  1.10 (0.97, 1.24)  1.13 (1.01, 1.29)  1.06 (1.01, 1.12)  —  1.17 (1.03, 1.32)  1.13 (1.00, 1.28)  1.23 (1.09, 1.40)  1.06 (1.01,1.11) | 0. 020  <0.001  <0.001  <0.001  0.026  0.033  <0.001  0.002  0.017  <0.001  <0.001  <0.001  0.318  0.128  0.048  0.012  0.014  0.053  0.001  0.010 |
| Model 1 was unadjusted. Model 2 was adjusted for age and sex. Model 3 was further adjusted for BMI, educational level, hypertension, diabetes, annual income, smoking status, drinking status, HDL-C, TG, RBC, creatinine, and TC.  Abbreviations: AISI, aggregate index of systemic inflammation; BMI, body mass index; CI, confidence interval; HDL-C, high-density lipoprotein cholesterol; HR, hazard ratio; NLR, neutrophil-to-lymphocyte ratio; NPAR, neutrophil percentage-to-albumin ratio; PLR, platelet-to-lymphocyte ratio; Q, quartile; RBC, red blood cell count; SII, systemic immune-inflammation index; SIRI, systemic inflammatory response index; TC, total cholesterol; TG, triglyceride. | | | | | | |
